# A cross-sectional study on spouse and parent differences in caregiving experiences of people living with schizophrenia in rural China

**DOI:** 10.1186/s12888-020-02633-w

**Published:** 2020-05-12

**Authors:** Yu Yu, Tong-xin Li, Yi-lu Li, Dan Qiu, Shi-jun Xi, Shui-yuan Xiao, Jacob Kraemer Tebes

**Affiliations:** 1 Hospital Evaluation Office, Xiangya Hospital, Central South University, Xiangya Road 87, Changsha, Hunan 410008 China; 2 Division of Prevention and Community Research, Department of Psychiatry, Yale School of Medicine, 389 Whitney Avenue, New Haven, CT 06511 USA; 3 Department of Social Medicine and Health Management, Xiangya School of Public Health, Central South University, Upper Mayuanlin Road 238, Changsha, Hunan 410008 China; 4 Mental Health Center, Xiangya Hospital, Central South University, Xiangya Road 87, Changsha, 410008 Hunan China

**Keywords:** Schizophrenia, Family caregivers, Caregiver burden, Caregiving, parent, spouse

## Abstract

**Background:**

Conflicting evidence exists on whether parent or spouse caregivers experience better outcomes when caring for family members with schizophrenia. The current study aims to examine relative caregiving experiences and impacts of spouse and parent caregivers for people living with schizophrenia (PLS) in China.

**Methods:**

A cross-sectional study was conducted in a sample of 264 community-dwelling primary family caregivers of PLS. Face-to-face interviews were conducted to collect information on family caregiving activities; negative caregiving impacts including objective and subjective burden, and caregiver psychological distress such as depression and anxiety; positive caregiving impacts including caregiving rewarding feelings, and family functioning for spouse and parent caregivers.

**Results:**

Both types of caregivers report engaging in similar caregiving activities and report comparable levels of objective burden. However, parent caregivers report significantly higher subjective burden than spouse caregivers (*b* = 7.94, 95%*CI*:2.08, 13.80, *P* < 0.01), which is also reflected in significantly higher depression (*b* = 3.88, 95%*CI*:1.35, 6.41, *P* < 0.01) and anxiety (*b* = 2.53, 95%*CI*: 0.22, 4.84, *P* < 0.05), and lower family functioning (*b* = − 1.71, 95%*CI*: − 2.73, − 0.49, *P* < 0.01). Despite these differences, both groups of caregivers report comparable rewarding feelings about caregiving.

**Conclusions:**

Our findings have implications for family caregivers globally, but especially for countries that adhere to Confucian cultural values and provide guidance for future family intervention programs. Such programs may do well to incorporate cultural values and beliefs in understanding caregiving and kinship family dynamics so as to support family caregivers, and in particular, the specific vulnerabilities of parent caregivers.

## Background

Across the world, schizophrenia is a debilitating, persistent psychiatric disorder that adversely affects individuals with the disorder as well as their family members who provide support and care for people living with schizophrenia (PLS) [1–3]. Such family caregivers often serve as an extension of the mental health system [4, 5]. It is now recognized that globally, family caregiving is likely to grow for PLS especially in lower-and middle-income countries with under-resourced mental health and social service systems [6]. In China, the number of PLS has increased significantly from 3.09 million in 1990 to 7.16 million in 2010 [7]; over 90% live with their family and receive family care due to insufficient community resources [8] .

Numerous studies across the world have shown various negative impacts of caregiving on caregivers, among which caregiver burden and psychological distress have been the most extensively studied and widely reported across cultures [5, 9]. Two types of burden have been identified: objective (e.g., manual tasks, household duties, etc.) and subjective (e.g., caregiver’s perception of burden, distress, stigma, etc.), and each may be experienced as physical, mental, financial, or social demands [5, 9]. Subjective burden has been found to be a major determinant of caregiver psychological distress, but objective burden, which usually depends on specific caregiving activities, appears not to have a direct influence on distress [10]. Psychologic distress has been a very common phenomenon among caregivers. Both qualitative and quantitative studies have shown depression and anxiety as the two most frequently reported psychological distress among caregivers [11–17]. Face-to-face qualitative interviews conducted with a diverse sample of U.S. caregivers of PLS showed that the most frequently reported impact of caring for a person with schizophrenia was feeling “emotional”, including feeling overwhelming, sad, frustrated, embarrassed, angry, stressed, anxious and worried; experiences that have also been described by caregivers with analogies like “rollercoaster” “white-water rafting” and “feeling underwater” [15]. Other quantitative studies consistently reported a high level of psychological distress for caregivers, with the prevalence of depression and anxiety at or above 80% [16, 17].

Although most of literature has described caregiving as having negative effects, it is increasingly recognized that caregiving may have positive influences on both the caregiver and family [18]. Caring for a family member with schizophrenia may bring about a positive transformation in a caregiver’s life and can promote positive inner feelings, such as improved self-admiration, self-affirmation, self-confidence, self-satisfaction and personal growth; referred to as caregiving rewarding feelings [10, 19]. In addition, caregiving may also impact family functional dynamics and relationships in a positive way, especially in Asian countries that attach great importance to familism and collectivism consistent with Confucian cultural values [20–24]. Research has identified various aspects of positive family functional dynamics in caregiving families, including family cohesion, family connectedness, family resilience and family hardiness, which may be generally referred to as family functioning [23, 24]. In well-functioning families, all family members will actively work together to manage concurrent demands and utilize available resources in adapting challenges and adversities to achieve a degree of balance [23].

Despite the voluminous amount of literature on the experiences and impacts of caregivers of PLS, there are several limitations in the previous studies. First, most research has not distinguished between objective and subjective burden. Second, limited research has been directed at examining specific caregiving activities as well as the positive impact of caregiving. Third, most caregiving research in mainland China has been conducted in urban areas using convenience sampling from hospitals, whereas research in rural communities is underrepresented even though rural caregiver experiences may vary from urban experiences due to differences in economy, education, family structure, and culture. And more importantly, most studies have not differentiated experiences and impacts among types of kinship caregivers, such as spouses and parents.

Identifying impacts of caregiving and the differences among family caregiver types may identify vulnerabilities that inform targeted interventions, but to date, evidence for such differences is mixed or limited [25–28]. Conflicting evidence exists on caregiver burden between spouses and parents. For instance, the World Health Organization World Mental Health Surveys [6] have shown higher caregiver burden for spouses than parents across high- and upper-middle-income countries, yet higher caregiver burden for parents than spouses from lower- or middle-income countries [29]. One study on Chinese caregivers of PLS found that parent caregivers reported higher burden than spouse caregivers [30], while another study of African caregivers of PLS showed no significant difference in caregiver burden between spouse and parent caregivers [31]. These results indicate that caregiver burden may be culture-dependent and vary by country. Comparison of psychological distress between spouse and parent caregivers showed inconsistent results. For instance, Chang et al. [32] compared both depression and anxiety between caregivers of individuals with serious mental illness (with most diagnosed with schizophrenia) in Taiwan but found no statistically significant differences between the two groups in either depression or anxiety. Similar results were reported in Stanley et al.’s [13] study on caregivers of PLS in India where no significant differences were observed based on parent/spouse status in depression and anxiety. However, another similar study conducted in Singapore found much lower scores in the psychological domain in spousal caregivers as opposed to parental caregivers [14]. Since research on the positive impact of caregiving such as rewarding caregiving feelings and family functioning is limited, even less is known about the relative positive impacts between parent and spouse caregivers.

In view of the wide range of caregiving impacts and inconclusive evidence on relative differences among kinship caregiver types, it is critical to examine a range of caregiving impacts between spouse and parent caregivers in a representative rural community of mainland China. The current study addresses this limitation by comparing caregiving activities, negative caregiving impacts including objective and subjective burden, and caregiver psychological distress such as depression and anxiety, positive caregiving impacts including caregiving rewarding feelings, and family functioning between rural spouse and parent caregivers. In the current study, spouses were defined as a wife or husband in marriage, as well as non-marital partners who have accepted a social role similar to that of a spouse, but do not have rights and duties reserved by law to a spouse; parents were defined as a biological or adoptive father or mother, with caregiving responsibilities. Based on conflicting evidence on comparisons between parent and spouse caregivers, we did not specify a hypothesis as to specific differences of each group or which group would be better or worse off, but rather conducted exploratory analyses to examine such differences.

## Methods

### Participants and procedure

This cross-sectional study was conducted in Ningxiang County of Hunan province from November 2015 to January 2016. Our sample was primary caregivers of PLS that were registered in the “Central Government Support for the Local Management and Treatment of Severe Mental Illnesses project”, also named as “686 program”, which is China’s largest demonstration project for mental health services that seeks to integrate hospital and community services for serious mental illness [33, 34]. The 686 Program provides multiple comprehensive mental health services including: patient registration and initial assessment, free medication and regular follow-up in the community, management of community emergencies, and free emergency hospitalization [33, 34].

A one-stage cluster-sampling method was used to recruit primary caregivers of PLS from the 686 Program. First, three Towns and 1 Xiang (an administrative unit similar to a town but with lower socio-economic development) were randomly selected from Ningxiang County, and then followed by whole sampling of all communities within each Town and all villages within each Xiang, leading to a total sampling frame of 55 representative communities/villages. This sampling method was used to obtain a sample as representative as possible and minimize sampling bias. A local primary care physician within each community/village assisted in identifying eligible primary caregivers based on a list of PLS registered in the 686 Program. Eligibility criteria for primary family caregivers included: 1) caring for a family member with schizophrenia who is registered in the 686 Program; 2) the family member being cared for meets criteria of the Chinese Classification of Mental Disorders-3 (CCMD-3) or the International Classification of Diseases-10 (ICD-10) for schizophrenia; 3) the primary caregiver is living with the PLS at least for the past two years and assumes major responsibilities for caregiving; 4) the primary caregiver is older than 16 years of age, since by Chinese civil law, people at the ages of 16 and older who are engaged in labor (here referred to as “caregiving”) and have a source of economic income are considered to have full capacity for civil conduct; and 5) the primary caregiver speaks Chinese, is literate, and not seriously disabled, thus enabling the person to understand and communicate in face-to-face interviews with the caregivers. Exclusion criteria included: 1) caring for family member with diagnosis other than schizophrenia such as depression and epilepsy; 2) primary family caregivers who do not speak Chinese or having serious physical or mental illness that makes them unable to communicate effectively in a face-to-face interview. A total of 352 primary family caregivers of PLS were identified in the final sample.

The community/village primary care physician visited each study household with a member of the research team to obtain informed consent from the caregivers, who then completed a series of questionnaires (see measures below) in face-to-face interviews, which were all conducted in Chinese. All caregivers were reimbursed with small gifts equivalent to RMB ¥ 10 (approximately USD$1.5) in return for their participation. Details of the subject recruitment process have been described elsewhere [35, 36].

Of the 352 caregivers approached, 327 participated in the interview (response rate: 93%). Among the 25 caregivers that refused the interview, reasons for refusal included no interest in the study (*n* = 11), concern about stigma (*n* = 9), too painful to share caregiving experiences (*n* = 3) and other reasons not disclosed (*n* = 2). No significant differences in socio-demographic background were observed between those who did or did not agree to participate based on age, gender, marital status, employment and education (available as Additional file 1). In the current study, only parents (*n* = 151) and spouses (*n* = 113) were included in the analyses, resulting in a final sample of 264. This sample size of 264 detected an effect size of 0.4 between parent and spouse caregivers by two-tailed t-test, assuming α = 0.05, β = 0.10.

### Measures

#### Socio-demographic information

Demographic information of both the PLS and the primary caregiver was collected by asking the primary caregivers on a questionnaire designed for this study, which included gender, age, marital status, occupation, education, family financial status, and kinship between PLS and the primary caregiver. For the primary caregivers, we also asked if there were any co-caregivers, dependents, physical illnesses, and how care had been provided for the PLS. This information was collected through face-to-face interviews between the research team and primary caregivers.

#### PLS functioning

PLS functioning was assessed using the Global Assessment of Functioning scale (GAF) [37]. The GAF is an axis of the DSM-IV and assessed the person’s overall social, occupational and psychological functioning in the past month from 1 (lowest) to 100 (highest), with examples provided for each ten-point interval. The GAF was first translated in Chinese by Zhang in 1984 [38] and proved to be a reliable and valid measure of psychiatric functioning [37, 38]. For the current study, PLS GAF was assessed by the research team based on a combination of caregivers’ report, direct assessment of the PLS, and a review of their clinical records, whenever available.

#### Caregiving activities

Specific caregiving activities were assessed by four “Yes-No” questions asking if the respondent was involved with the following four aspects of caregiving activities for the PLS: daily activities (e.g., eating, drinking, getting dressed), medication management (e.g., monitoring medication, buying medicine), hospital visits (e.g., registration, hospitalization), and financial help (e.g., spending and giving money). Responses of “no” were scored as 0, and “yes” as 1, and then followed by asking how often the respondent was involved with each caregiving activity, ranging from “occasionally” (scored as 1) to “always” (scored as 4). Detailed information about the questions and optional answers can be found in the Additional file 2. For the current study, questions on caregiving activities were administered by the research team to primary caregivers.

#### Objective burden

Objective burden was assessed using the Family Burden Interview Schedule (FBIS) [39] classified into six categories: financial burden, disruption of routine family activities, family leisure, family interactions, and effect on physical and mental health of others. The scale consists of 24 items rated on a 3-point Likert scale from 0 (no burden) to 2 (serious burden). The total score ranges from 0 to 48 with higher scores showing higher family burden. The FBIS was first translated into Chinese by Chien et al. [40] in 2004 and proved to be a reliable and valid measure of objective family burden. For the present study, FBIS was administered by the research team to primary caregivers and the Chinese version of FBIS showed acceptable internal consistency with a Cronbach’s α of 0.86 for the total scale and ranged from 0.63 to 0.86 for the subscales.

#### Subjective burden

Subjective burden was assessed using the Zarit Burden Interview (ZBI) [41]. The ZBI consists of 22 items scored on a 5-point Likert scale from 0 (never) to 4 (nearly always), except for the final item on global burden, rated from 0 (not at all) to 4 (extremely). The total score ranges from 0 to 88 with higher scores indicating higher perceived burden [42, 43]. The ZBI was first translated into Chinese by Lu et al. [44] in 2009 and proved to be a reliable and valid measure of subjective caregiver burden. For the present study, ZBI was administered by the research team to primary caregivers and the Chinese version of ZBI showed acceptable internal consistency with a Cronbach’s α coefficient of 0.89.

#### Caregiver depression

Caregiver depression was measured using the 9-item Patient Health Questionnaire (PHQ-9) [45], which consists of 9 items scored in 4-point Likert scale from 0 (not at all) to 3 (nearly every day). The total score ranges from 0 to 27, with higher scores implying more depressive symptoms, and a cut-off point of 10 differentiating depression and non-depression [46, 47]. The PHQ-9 was first translated into Chinese by Yeung et al. [48] in 2008 and proved to be a reliable and valid measure of depression. For the present study, PHQ-9 was administered by the research team to primary caregivers and the Chinese version of the PHQ-9 demonstrated good internal consistency with a Cronbach’s α coefficient of 0.89.

#### Caregiver anxiety

Caregiver anxiety was measured by the 7-item Generalized Anxiety Disorder Scale (GAD-7) [49] which detects the primary caregiver’s anxiety symptoms during the past two weeks. Each item is rated on a 4-point Likert scale from 0 (not at all) to 3 (nearly every day). The total score ranges from 0 to 21, with a cut-off point of 10 differentiating anxiety and non-anxiety [50]. The GAD-7 was first translated into Chinese by He et al. [51] in 2010 and proved to be a reliable and valid measure of anxiety. For the present study, GAD-7 was administered by the research team to primary caregivers and the Chinese version of the GAD-7 demonstrated good internal consistency in the current study with a Cronbach’s α coefficient of 0.91.

#### Caregiving rewarding feelings

Positive feelings about caregiving were assessed using the caregiving rewarding feelings (CRF) scale which was designed for this study. CRF was initially developed based on qualitative interviews on a convenience sample of 30 pairs of PLS and their primary caregivers, then revised based on pre-testing and Delphi’s method, and validated in a larger sample. The detailed development and validation process of the CRF has been described elsewhere [52]. The CRF was developed in Chinese and consists of 12 items asking about a range of possible rewarding feelings that caregivers may have during caregiving. Each “yes” response was scored 1 and “no” response 0, with total scores ranging from 0 to 12; higher scores indicate more positive feelings in caregiving. For the present study, CRF was administered by the research team to primary caregivers and the Chinese version of CRF showed acceptable reliability with a Cronbach’s alpha of 0.77.

#### Caregiver perceived family functioning

Caregiver perceived family functioning was assessed using the Family Adaptation, Partnership, Growth, Affection and Resolve Index scale (APGAR) [53]. It consists of 5 items scored in 3-point Likert scale from 0 (hardly ever) to 2 (almost always). The total score ranges from 0 to 10 with higher scores indicating higher satisfaction with family functioning. The APGAR was first translated into Chinese by Chang et al. [54] in 1993 and proved to be a reliable and valid measure of family functioning. For the present study, APGAR was administered by the research team to primary caregivers and the Chinese version of APGAR showed acceptable internal consistency with a Cronbach’s α coefficient of 0.91.

### Statistical analysis

Data analyses examined missing values, influential values and outliers, skewness, and kurtosis. Frequencies and percentages were displayed for categorical variables, and means with standard deviations or medians with interquartile ranges (IQR) displayed for continuous variables. We compared socio-demographic characteristics, caregiving activities, and six types of caregiving impacts (objective family burden, subjective caregiver burden, depression, anxiety, caregiving rewarding feelings, and family functioning) between spouse and parent caregivers using two-group tests. Depending on variable type and data distribution, we conducted various two-group tests including: independent two sample t-tests for normally distributed continuous data, Mann-Whitney U test for non-normally distributed continuous data, and Pearson’s chi-square tests for categorical variables. In order to examine the predictive effect of kinship (spouse vs parent) on the six types of caregiving impacts, we further conducted six separate multivariate linear regressions, with kinship (spouse vs parent) as independent variables, and the six types of caregiving impacts as dependent variables, while controlling for all potential confounders (PLS functioning, gender, and education, as well as caregiver age, gender, marriage, employment, education, family income, whether have co-caregivers, whether there were additional dependents, whether caregivers have physical illness, and length of caring) to avoid analysis bias (Table 4). Data were analyzed using STATA software version 15.0, with *p* values smaller than 0.05 in two-tailed tests considered as statistically significant.

### Ethical considerations

This study was approved by the Ethics Review Committee of the Xiangya School of Public Health of Central South University in China. The interviewed participants were informed verbally and in writing of the study’s purpose, their right to refuse to participate, and the voluntary nature of their participation. All participants provided written informed consent before the interviews.

## Results

### Group comparison of sample characteristics

Table 1 describes characteristics of the PLS. The median age was 44 for the PLS. Slightly less than half of PLS were female, married, with a primary education, and most were unemployed. PLS functioning as measured by the GAF showed a median score of 42, indicating serious impairment of functioning. We further compared the socio-demographic characteristics of PLS cared for by spouses and parents. Compared to PLS cared for by a spouse, PLS cared for by a parent had lower functioning (median: 35 vs 55), were younger (median: 38 vs 53), were more likely to be male (70% vs 29%), were less likely to be married (76% vs 100%) and had higher educational attainment (66% vs 42%).

**Table 1:** Socio-demographic characteristics of PLS and comparison between PLS cared for by spouses and parents

Variables		Kinship types ***N*** (%)/ Median (IQR)			***Z/χ2***	P^*^
		Total	Cared by Spouses (***n*** = 113)	Cared by Parents(***n*** = 151)		
**Functioning (GAF)**	Median (IQR)	42 (5–90)	55 (11–85)	35 (5–85)	3.952	**< 0.001**
**Age**	Median (IQR)	44 (23–72)	53 (35–72)	38 (23–53)	10.422	**< 0.001**
**Gender**	Male	138 (52.27)	33 (29.20)	105 (69.54)		
	Female	126 (47.73)	80 (70.80)	46 (30.46)	42.143	**< 0.001**
**Marriage**	Married	115 (43.56)	113 (100)	115 (76.16)		
	Not married	149 (56.44)	0 (0)	36 (23.84)	152.481	**< 0.001**
**Employment**	Employed	41 (15.53)	14 (12.39)	27 (17.88)		
	Not employed	223 (84.47)	99 (87.61)	124 (82.12)	1.486	0.223
**Education**	Primary	116 (43.94)	65 (57.52)	51 (33.77)		
	Middle	99 (37.50)	31 (27.43)	68 (45.03)		
	High	49 (18.56)	17 (15.04)	32 (21.19)	14.950	**< 0.001**

*GAF* Global Assessment of Function scale ^*^numbers in bold represents significant values at *P* = 0.05/0.01

Table 2 describes characteristics of the primary caregivers. The median age was 62 for the primary caregivers. Slightly more than half of caregivers were female, employed, without co-caregivers and with additional dependents. Also, most caregivers were married, of a primary education^1^ and with some physical illness and had been caring for the PLS for many years (median = 15 years). We also compared socio-demographic characteristics between spouse and parent caregivers. Compared to spouse caregivers, parent caregivers themselves were older (median: 64 vs 57) and had fewer years of caregiving (median: 14 vs 19); were more likely to be female (70% vs 32%), had co-caregivers (52% vs 28%) and had a physical illness (78% vs 67%); they were also less likely to be married (67% vs 100%), employed (44% vs 61%) and with middle and high school education (24% vs 42%).

**Table 2:** Socio-demographic characteristics of caregivers and comparison between spouses and parents

Variables		Kinship types ***N*** (%)/ Median (IQR)			***Z/χ2***	***P***
		Total	Spouse (***n*** = 113)	Parents (***n*** = 151)		
**Age**	Median (IQR)	62 (41–80)	57 (41–74)	64 (45–80)	−6.013	**< 0.001**
**Gender**	Male	123 (46.59)	77 (68.14)	46 (30.46)		
	Female	141 (53.41)	36 (31.86)	105 (69.54)	43.069	**< 0.001**
**Marriage**	Married	214 (81.06)	113 (100.00)	101 (66.89)		
	Not married	50 (18.94)	0 (0)	50 (33.11)	46.160	**< 0.001**
**Occupation**	Employed	136 (51.52)	69 (61.06)	67 (44.37)		
	Not employed	128 (48.48)	44 (38.94)	84 (55.63)	7.209	**0.007**
**Education**	Primary	179 (67.80)	65 (57.52)	114 (75.50)		
	Middle	59 (22.35)	35 (30.97)	24 (15.89)		
	High	26 (9.85)	13 (11.50)	13 (8.61)	10.206	**0.006**
**Family income** (Yuan/ person.year)	Median (IQR)	3750 (2000–7450)	5000 (1920–8000)	3333 (2000–6667)	1.329	0.184
**Co-caregivers**	No	153 (57.95)	81 (71.68)	72 (47.68)		
	Yes	111 (42.05)	32 (28.32)	79 (52.32)	15.277	**< 0.001**
**Additional dependents**	No	129 (48.86)	60 (53.10)	69 (45.70)		
	Yes	135 (51.14)	53 (46.90)	82 (54.30)	1.417	0.234
**Physical illness**	No	70 (26.52)	37 (32.74)	33 (21.85)		
	Yes	194 (73.48)	76 (67.26)	118 (78.15)	3.933	**0.047**
**Length of caring (year)**	Median (IQR)	15 (1–45)	19 (2–43)	14 (2–39)	3.049	**0.002**

*GAF* Global Assessment of Function scale *numbers in bold represents significant values at *P* = 0.05/0.01

### Group comparison of caregiving activities

Table 3 shows the type and frequency of care provided by the primary caregivers to the PLS. Generally, most caregivers were actively involved with all four types of caregiving tasks, with participation rate ranging from 61% for financial help to 73% for medication management. For the frequency of each caregiving activity, a median frequency of 4 (always) was reported for all caregiving types except for daily activities (median frequency = 3). This difference may be because PLS in this sample had few physical disabilities that required assistance in daily activities as opposed to assistance with medication management, hospital visits, and finances.

**Table 3:** Caregiving activity involvement of parent and spouse caregivers

Variables		Kinship types ***N*** (%)/ Median (IQR)			***Z/χ2***	P*
		Total	Spouse(***n*** = 113)	Parent(***n*** = 151)		
**Daily activities**	**No**	103 (39.02)	45 (39.82)	58 (38.41)		
	**Yes**	161 (60.98)	68 (60.18)	93 (61.59)	0.0542	0.816
	**Frequency**	3 (0–4)	3 (0–4)	4 (0–4)	−0.671	0.502
**Medicine management**	**No**	70 (26.52)	28 (24.78)	42 (27.81)		
	**Yes**	194 (73.48)	85 (75.22)	109 (72.19)	0.306	0.580
	**Frequency**	4 (0–4)	4 (0–4)	4 (0–4)	0.330	0.742
**Hospital visit**	**No**	78 (29.55)	33 (29.20)	45 (29.80)		
	**Yes**	186 (70.45)	80 (70.80)	106 (70.20)	0.0111	0.916
	**Frequency**	4 (0–4)	4 (0–4)	4 (0–4)	0.187	0.851
**Financial help**	**No**	104 (39.39)	40 (35.40)	64 (42.38)		
	**Yes**	160 (60.61)	73 (64.60)	87 (57.62)	1.321	0.250
	**Frequency**	4 (0–4)	4 (0–4)	3 (0–4)	1.042	0.298

* All *P* values were non-significant with values above 0.05

We also found no statistically significant difference between spouse and parent caregivers in their involvement in or the frequency of these four types of caregiving activities, indicating that parents and spouses were providing similar types of care for their loved one with schizophrenia.

### Group comparisons of caregiving impact

Table 4 shows six types of caregiving impacts that includes both negative and positive impacts. Negative impacts include both objective family burden as measured by FBIS (median = 26) and subjective caregiver burden as measured by ZBI (mean = 44), as well as psychological distress including depression and anxiety, with a median of 9 for both. Positive impacts include caregiving rewarding feelings as measured by CRF (median = 7) and family functioning as measured by APGAR (median = 6).

**Table 4:** Caregiving impacts between spouse and parent caregivers

Variables	Kinship types ***N*** (%)/ Median (IQR)/Mean ± SD			***z/t/χ***^***2***^	***P***
	Total	Spouse(***n*** = 113)	Parents(***n*** = 151)		
**Family Burden (FBIS)**	26 (3–43)	22 (4–39)	27 (6–42)	−2.56	**0.011**
**Caregiver burden (ZBI)**	43.96 ± 18.25	40.81 ± 18.13	46.18 ± 18.07	− 2.24	**0.026**
**Depression (PHQ-9)**	9 (5–16)	6.5 (3–12)	12 (6–17)	−3.95	**< 0.001**
**Anxiety (GAD-7)**	9 (5–15)	7 (2–13)	11 (6–17)	−3.54	**< 0.001**
**Caregiving rewarding feelings (CRF)**	7 (5,9)	7 (5,10)	7 (5,9)	1.74	0.083
**Family functioning (APGAR)**	6 (4–9)	8 (5,10)	6 (3,9)	3.10	**0.002**

*FBIS* Family Burden Interview Schedule; *ZBI* Zarit Burden Interview; *PHQ-9* 9-item Patient Health Questionnaire; *GAD-7* 7-item Generalized Anxiety Disorder Scale; *CRF* Caregiving Rewarding Feelings; *APGAR* Family Adaptation, Partnership, Growth, Affection and Resolve Index scale

A further comparison of the six types of caregiving impact between spouse and parent caregivers showed statistically significant differences in all except for caregiving rewarding feelings. Compared to spouse caregivers, parent caregivers reported higher objective family burden (27 vs 22, *P* = 0.011) and subjective caregiver burden (46.18 vs 40.81, *P* = 0.026), they also reported higher depression (12 vs 6.5, *P* < 0.001), anxiety (11 vs 7, *P* < 0.001) and lower family functioning (6 vs 8, *P* = 0.002).

### Multiple multivariate linear regressions of kinship on caregiving impacts.

Table 5 shows the results of six separate multivariate linear regressions of kinship (spouse vs parent) on the six types of caregiving impacts (family burden, caregiver burden, depression, anxiety, caregiving rewarding feelings, and family functioning), while controlling for potential confounders (PLS functioning, gender and education, and caregiver age, gender, marriage, employment, education, family income, whether have co-caregivers, whether there are additional dependents, whether caregivers have physical illness, and length of caring). Although no statistically significant differences in objective family burden (FBIS) were found between spouse and parent caregivers, parent caregivers reported much higher subjective caregiver burden, with a coefficient as high as 7.94 (95% *CI*: 2.08, 13.80). Also, multivariate analyses showed that parent caregiving independently and significantly predicted higher depression (*b* = 3.88, 95% *CI*: 1.35, 6.41) and anxiety (*b* = 2.53, 95% *CI*: 0.22, 4.84). For positive impacts of caregiving, although no statistically significant difference in caregiving rewarding feelings were found between spouse and parent caregivers, parent caregivers reported significantly lower family functioning with a coefficient of − 1.71 (95% *CI*: − 2.85, − 0.57).

**Table 5:** Multiple multivariate linear regressions of kinship predicting caregiving impacts

Variables				Coefficients	(95% CI)		
		Family burden (FBIS)	Caregiver burden (ZBI)	Depression (PHQ-9)	Anxiety (GAD-7)	Caregiving rewarding feelings (CRF)	Family functioning (APGAR)
**Kinship**	Spouse	ref	ref	ref	ref	ref	ref
	Parent	1.05 (−2.01, 4.11)	7.94^**^ **(2.08, 13.80)**	3.88^**^ **(1.35, 6.41)**	2.53^*^ **(0.22, 4.84)**	−0.69 (−1.72, 0.33)	−1.71^**^ **(− 2.85, − 0.57)**
**PLS functioning (GAF)**		−0.19^***^ **(− 0.24, − 0.14)**	− 0.36^***^ **(− 0.45, − 0.27)**	−0.09^***^ **(− 0.13, − 0.05)**	−0.05^**^ **(− 0.08, − 0.01)**	0.03^***^ **(0.01, 0.05)**	0.04^***^ **(0.02, 0.06)**
**PLS gender**	Male	ref	ref	ref	ref	ref	ref
	Female	0.56 (− 1.93, 3.06)	3.00 (− 1.78, 7.78)	0.02 (− 2.04, 2.08)	−0.44 (− 2.35, 1.46)	0.42 (− 0.41, 1.26)	−0.31 (− 1.23, 0.62)
**PLS education**	Primary	ref	ref	ref	ref	ref	ref
	Middle	−0.41 (− 2.93, 2.10)	−0.10 (−4.82, 4.80)	− 1.91 (−3.99, 0.17)	− 1.06 (− 2.97, 0.85)	−0.58 (− 1.42, 0.27)	−0.09 (− 1.03, 0.85)
	High	0.71 (− 2.42, 3.84)	− 0.36 (−6.42, 5.70)	−0.51 (− 3.08, 2.06)	−0.64 (− 3.00, 1.72)	−0.18 (− 1.22, 0.87)	−0.08 (− 1.24, 1.08)
**Caregiver age**		−1.51^*^ **(− 0.29, − 0.01)**	−0.42^**^ **(− 0.68, − 0.16)**	−0.11 (− 0.22, 0.01)	−0.01 (− 0.12, 0.09)	0.02 (− 0.03, 0.06)	0.03 (− 0.02, 0.08)
**Caregiver gender**	Male	ref	ref	ref	ref	ref	ref
	Female	2.77 (−0.01, 5.55)	−0.29 (−5.60, 5.02)	0.46 (− 1.82, 2.74)	1.80 (− 0.29, 3.89)	1.42^**^ **(0.50, 2.35)**	0.67 (− 0.36, 1.70)
**Caregiver marriage**	Married	ref	ref	ref	ref	ref	ref
	Not married	−1.43 (− 4.80, 1.94)	−6.72 (− 13.12, −0.32)	− 2.02 (− 4.77, 0.73)	−2.72 (− 5.27, − 0.17)	−0.40 (− 1.53, 0.72)	0.57 (− 0.68, 1.81)
**Caregiver employment**	Employed	ref	ref	ref	ref	ref	ref
	Not employed	0.59 (− 1.77, 2.95)	2.90 (− 1.65, 7.45)	0.60 (− 1.34, 2.55)	−0.28 (− 2.06, 1.51)	− 0.49 (− 1.28, 0.30)	−0.36 (− 1.24, 0.51)
**Caregiver education**	Primary	ref	ref	ref	ref	ref	ref
	Middle	1.04 (− 1.70, 3.78)	0.10 (−5.16, 5.35)	−0.46 (−2.71, 1.80)	− 0.72 (− 2.79, 1.35)	−0.26 (− 1.18, 0.65)	0.84 (− 0.18, 1.86)
	High	− 3.26 (− 6.97, 0.45)	−5.41 (− 12.53, 1.72)	−3.53^*^ **(− 6.58, − 0.48)**	−4.23 (−7.03, − 1.43)	1.14 (− 0.10, 2.38)	0.53 (− 0.85, 1.91)
**Caregiver family income**		−0.00 (− 0.00, 0.00)	−0.00 (− 0.00, 0.00)	−0.00 (− 0.00, 0.00)	−0.00 (− 0.00, 0.00)	−0.00 (− 0.00, 0.00)	−0.00 (− 0.00, 0.00)
**Co-caregivers**	No	ref	ref	ref	ref	ref	ref
	Yes	0.24 (− 2.14, 2.61)	−4.96^*^ **(−9.50, −0.43)**	−0.48 (− 2.43, 1.47)	−0.85 (− 2.65, 0.95)	0.16 (− 0.63, 0.95)	1.22^**^ **(0.34, 2.10)**
**Additional dependents**	**No**	ref	ref	ref	ref	ref	ref
	**Yes**	2.13 (−0.14, 4.40)	−0.83 (− 5.19, 3.53)	0.20 (− 1.67, 2.07)	−0.17 (− 1.89, 1.55)	0.40 (− 0.36, 1.16)	0.32 (− 0.52, 1.16)
**Physical illness**	**No**	ref	ref	ref	ref	ref	ref
	**Yes**	1.88 (−0.60, 4.37)	3.17 (−1.61, 7.94)	3.03^**^ **(0.96, 5.09)**	2.05^*^ **(0.15, 3.94)**	0.29 (−0.54, 1.12)	0.09 (−0.84, 1.01)
**Length of caring**		0.07 (−0.05, 0.19)	0.14 (−0.09, 0.37)	0.01 (− 0.09, 0.11)	−0.04 (− 0.13, 0.05)	0.01 (− 0.02, 0.05)	−0.02 (− 0.06, 0.03)
**R**^**2**^		34.44%	33.56%	24.47%	18.82%	13.84%	18.50%
**Mean VIF (range)**^**&**^		1.43 (1.07, 2.13)	1.42 (1.07, 2.12)	1.42 (1.07, 2.14)	1.43 (1.07, 2.13)	1.42 (1.07, 2.13)	1.42 (1.07, 2.13)

*P < 0.05, ** *P* < 0.01, *** *P* < 0.001, numbers in bold represents significant values at *P* = 0.05/0.01/0.001

*GAF* Global Assessment of Function; *PLS* People living with schizophrenia; *FBIS*: Family Burden Interview Schedule; *ZBI* Zarit Burden Interview; *PHQ-9* 9-item Patient Health Questionnaire; *GAD-7* 7-item Generalized Anxiety

Disorder Scale; *CRF* Caregiving Rewarding Feelings; *APGAR* Family Adaptation, Partnership, Growth, Affection and Resolve Index scale

^&^*VIF* variance inflation factor, indicating collinearity if VIF > 5, here all VIF are below the criteria thus excluding multicollinearity

### Post-hoc power analysis

We did a post-hoc power analysis based on one primary outcome of subjective caregiver burden measured by Zarit Burden Interview (ZBI). Assuming α = 0.05, the sample size of 113 for spouse caregivers (mean: 40.81) and 151 parent caregivers (mean: 46.18) were able to detect a significant difference with 95% power.

## Discussion

This study compared the caregiving experiences of spouse and parent caregivers of PLS in China. The results show that both types of caregivers – parents and spouses -- report engaging in similar caregiving activities and comparable levels of objective burden. However, parent caregivers report significantly higher subjective burden, which is reflected in significantly higher depression and anxiety scores, and in reports of lower family functioning. Despite these differences, both groups of caregivers report comparable rewarding feelings about caregiving.

As far as we know, no studies on caregiving of PLS have examined the specific caregiving activities of primary caregivers, a gap addressed by the current study through its examination of four specific types of caregiving activities: daily activities, medication management, hospital visits, and financial help. Our findings showed that parent and spouse caregivers are equally engaged in these activities, which is suggested by comparable reports of objective burdens across kinship caregivers. Our results are consistent with the extensive literature showing caregiving to be a highly-demanding experience that requires family caregivers to devote considerable time, energy and resources to provide round-the-clock care to a PLS, regardless of the specific activity [4, 5, 8, 9].

However, parent caregivers reported significantly higher subjective burden than spouse caregivers, even though objective burden and caregiver activities were both comparable. This is an important finding because it suggests that objective burden and what caregivers actually do may not fully reflect the subjective burden of what they actually feel. The consistent relationship between subjective burden and depression and anxiety scores also suggests a potential role for subjective burden in mediating the relationship between caregiving and health [4, 55]. Future longitudinal research should examine the relationship between subjective burden and health as well as the role of social supports in this relationship.

Another intriguing issue raised by the present study is the intersection of family caregiving with the dominant belief in collectivism that is deeply rooted in Chinese culture. Family members in China attach great importance to specific roles and proper relationships among family members, thus maintaining family cohesion, harmony, and equilibrium [5, 56]. As a result, different family members may perform similar caregiving tasks and experience similar objective burden since they are living in the same household and caring for the same person, yet their personal perception of burden may vary greatly due to the different role expectation they experience. For instance, parents, especially mothers, are more likely to be blamed for a child’s sickness since they are expected to give birth to healthy children and nurture them through their parenting role [57]. A child with schizophrenia may contribute to a marriage crisis and family conflict [18, 57, 58]. The mother and father may blame each other for the child’s condition, disagree about treatment plans, and argue about caregiving responsibilities. As a result, parents may perceive higher burden when caring for PLS due to the sense of guilt, blame, and anger that results from their situation [18, 57, 58]. In contrast, spouse caregivers may normalize caregiving as part of their marital role as reflected in marriage vows, such as “in sickness and in health” or “until death do us part” [59–61]. Spouses may thus experience less conflict between their caregiver roles and other family and work-related roles they have, and thus experience less subjective burden [62, 63]. Differences in subjective burden between spouse and parent caregivers may also be related to differences in perceived obligation, duty, and responsibility for supporting an individual with mental health problems. For example, spouse caregivers may feel less of a keen sense of duty or obligation to care for a PLS than parents who may feel they have no choice but to continue caregiving. Future research should examine directly the role of culture and family caregiving for PLS.

Related to the above issue is that parents reported being worse-off than spouse caregivers in both negative and positive caregiving impacts. Parents reported more perceived burden, more depression and anxiety symptoms, and lower perceived family functioning. These findings are consistent with the unique family-oriented culture common in Asian countries that emphasizes family cohesion and harmony, with special focus on familism and filial piety [20, 56, 64]. Chinese society is especially recognized for its strong sense of filial obligation, defined as: “the concept, desire, and behaviors of carrying on family line, being good, and obedient to, as well as taking care of one’s parents”. One key ideology of filial piety in China is the concept that “Bu Xiao You San, Wu Hou Wei Da” (There are three types of impieties, among which having no descendent is the worst) and “to have no posterity is unfilial” [65]. In a Chinese society that is based on close-knit family relationships, children are viewed as the only continuation of the family bloodline, thus representing the future prosperity of the entire family [66–68]. Children are expected to take care of their parents when they are old and frail [66–68], but a child with schizophrenia may mean that the family bloodline may be broken and that the parents have no one to depend on when they grow old. The situation is accentuated with the “one child per family” policy that persisted for over three decades before it was recently abolished [69].

## Limitations

This study has several limitations. First, its cross-sectional design limits being able to examine differences among spouse and parent caregivers over time. Further longitudinal research is needed to look at such caregiving experiences and impacts, as family caregivers’ views of their responsibilities for a mentally ill relative do evolve over time [70]. Second, many caregivers had been caring for their family members with schizophrenia for a long time, and thus the experiences of this sample may be different from others caring for a PLS after a first episode. Future research may seek to examine this issue. Third, although our total sampling frame included various kinships including spouse, parents, siblings, children and others, we only were able to examine spouse and parent caregivers due to an insufficient sample size to examine other kinship types. Future research should consider examining differences across more diverse caregiver types. Fourth, participants were approached through the 686 Program and thus excluding caregivers of PLS not registered into the system. It is likely that caregivers of PLS outside the 686 Program may have different caregiving impacts due to lack or lower quality of needed services. Future research may seek to include the caregivers of PLS outside the 686 Program to get a more representative picture of caregiving experiences and impacts for all PLS in China.

## Conclusions

This study found that parent caregivers of PLS reported significantly higher subjective burden, depression, anxiety, and lower family functioning than spouse caregivers, even though both types of caregivers reported comparable caregiving activities, objective burden, and caregiving rewarding feelings.

These results have several implications. First, caregiver intervention programs that target individual caregivers may need to address the specific vulnerabilities of parent caregivers of PLS, such as increased subjective burden, depression, and anxiety, and reduced family functioning. Second, family caregiver intervention programs should take into account kinship family dynamics when supporting family caregivers of PLS. One promising approach may be to offer psychoeducation to family members of the PLS as part of ongoing treatment and rehabilitation [71–73]. And third, this study suggests that it may be particularly useful to incorporate cultural values and beliefs in understanding the family processes at work for family caregivers of PLS. Although our study was conducted in mainland China, our findings are relevant to other countries, especially those that share Confucian beliefs common to Asian culture.

## Supplementary information

**Additional file 1.** Comparison of socio-demographics between respondents and non-respondents. Showing analysis results of comparison between the two groups.

**Additional file 2.** Caregiving experience. Showing items and optional answers of the self-designed caregiving experience scale.

## Data Availability

The datasets used and/or analyses are available from the corresponding author with a reasonable request.

## Abbreviations

PLS: People Living with Schizophrenia
CCMD-3: Chinese Classification of Mental Disorders-3
ICD-10: International Classification of Diseases-10
GAF: Global Assessment of Function
FBIS: Family Burden Interview Schedule
ZBI: Zarit Burden Interview
PHQ-9: 9-item Patient Health Questionnaire
GAD-7: 7-item Generalized Anxiety Disorder
CRF: Caregiving Rewarding Feelings
APGAR: Family Adaptation, Partnership, Growth, Affection and Resolve index scale
IQR: Interquartile Ranges

## Footnotes

1 Primary school goes through grade 6 in China.
