# Supplementary material for: A cross-sectional study on spouse and parent differences in caregiving experiences of people living with schizophrenia in rural China
Source: BMC Psychiatry. 2020 May 12;20:226. doi: 10.1186/s12888-020-02633-w (PMC7216408; doi:10.1186/s12888-020-02633-w)
Supplement: Supplementary file 1 — Additional file 1. Comparison of socio-demographics between respondents and non-respondents. Showing analysis results of comparison between the two groups. [file 12888_2020_2633_MOESM1_ESM.docx]

**Appendix S1. Comparison of socio-demographics between respondents and non-respondents**

| **Variables** |  | **Response (n=327)** | **Non-response (n=25)** | ***Z/χ2*** | ***P*** |
| --- | --- | --- | --- | --- | --- |
| **Age** | Median (IQR) | 59 (17, 81) | 59 (18, 70) | -0.131 | 0.896 |
| **Gender** | Male | 151 (46.2) | 12 (48.0) |  |  |
|  | Female | 176 (53.8) | 13 (52.0) | 0.031 | 0.860 |
| **Marriage** | Married | 269 (82.3) | 20 (80.0) |  |  |
|  | Not married | 58 (17.7) | 5 (20.0) | 0.081 | 0.776 |
| **Employment** | Employed | 173 (52.9) | 12 (48.0) |  |  |
|  | Not employed | 154 (47.1) | 13 (52.0) | 0.224 | 0.636 |
| **Education** | Primary | 196 (59.9) | 16 (64.0) |  |  |
|  | Middle | 87 (26.6) | 7 (28.0) |  |  |
|  | High | 44 (13.5) | 2 (8.0) | 0.610 | 0.737 |
