# Supplementary material for: A cross-sectional study on spouse and parent differences in caregiving experiences of people living with schizophrenia in rural China
Source: BMC Psychiatry. 2020 May 12;20:226. doi: 10.1186/s12888-020-02633-w (PMC7216408; doi:10.1186/s12888-020-02633-w)
Supplement: Supplementary file 2 — Additional file 2. Caregiving experience. Showing items and optional answers of the self-designed caregiving experience scale. [file 12888_2020_2633_MOESM2_ESM.docx]

**Appendix S2 Caregiving experience**

- 1. Are you involved in taking care of the patient’s daily activities, such as eating, drinking, getting dressed, getting a shower, going to the toilet, getting outside?

① Yes ② No

- 1. If yes, how often are you involved in taking care of the patient’s daily activities?

① Occasionally ② Sometimes ③ Often ④ always

- 1. Are you involved in managing the patient’s medicine, such as monitoring medication, helping with buying and getting the medicine, etc.?

① Yes ② No

2.1 If yes, how often are you involved in managing the patient’s medicine?

① Occasionally ② Sometimes ③ Often ④ always

- 1. Are you involved in helping with the patient’s hospital visit, such as taking the patient to the doctor, helping with registration, hospitalization, etc.?

① Yes ② No

3.1 If yes, how often are you involved in helping with the patient’s hospital visit?

① Occasionally ② Sometimes ③ Often ④ always

- 1. Are you involved in providing financial support to the patient’s, such as spending money on the patient, giving money to the patient, etc.?

① Yes ② No

4.1 If yes, how often are you involved in providing financial support to the patient?

① Occasionally ② Sometimes ③ Often ④ always
